# Supplementary material for: A Statistical Perspective on Transformers for Small Longitudinal Cohort Data
Source: Stat Med. 2026 Sep 23;45(23-24):e70744. doi: 10.1002/sim.70744 (PMC13600780; doi:10.1002/sim.70744)
Supplement: Supplementary file 1 [file SIM-45-0-s001.pdf]

SUPPORTING INFORMATION

S1 | EMPIRICAL NULL CALIBRATION OF THE PERMUTATION TEST

To probe the null behavior of the permutation test in Section 2.3 beyond the small number of repetitions used in the main text, we increased the number of repetitions to 500 and for each variable, recorded the empirical distribution of p-values across repetitions. We used the simulation of Section 3.1 with $n_{\text{train}} = 200$ and visit-sample size $V = 5$, predicting variable j_3.

Figure S1 shows the per-variable p-value histograms. Signal variables (j_1, j_2, j_3 in red) place a large proportion of their p-values near zero, in line with the test's intended power. Null variables (in blue) place essentially no mass below 0.05: the empirical Type-I error rate at $\alpha = 0.05$ is exactly 0.000 across all seven null variables and all 500 repetitions, i.e. across 3500 trials. Their distributions are concentrated in $[0.3, 0.8]$ rather than uniform on $[0, 1]$, that is the conservative shift predicted in Section 2.3. Power on the three signal variables is 0.88, 0.44 and 0.71 at $\alpha = 0.05$, with mean 0.677. Figure S2 confirms the same pattern via quantile-quantile plots: null-variable curves sit systematically above the 45° diagonal, while signal-variable curves fall below it.

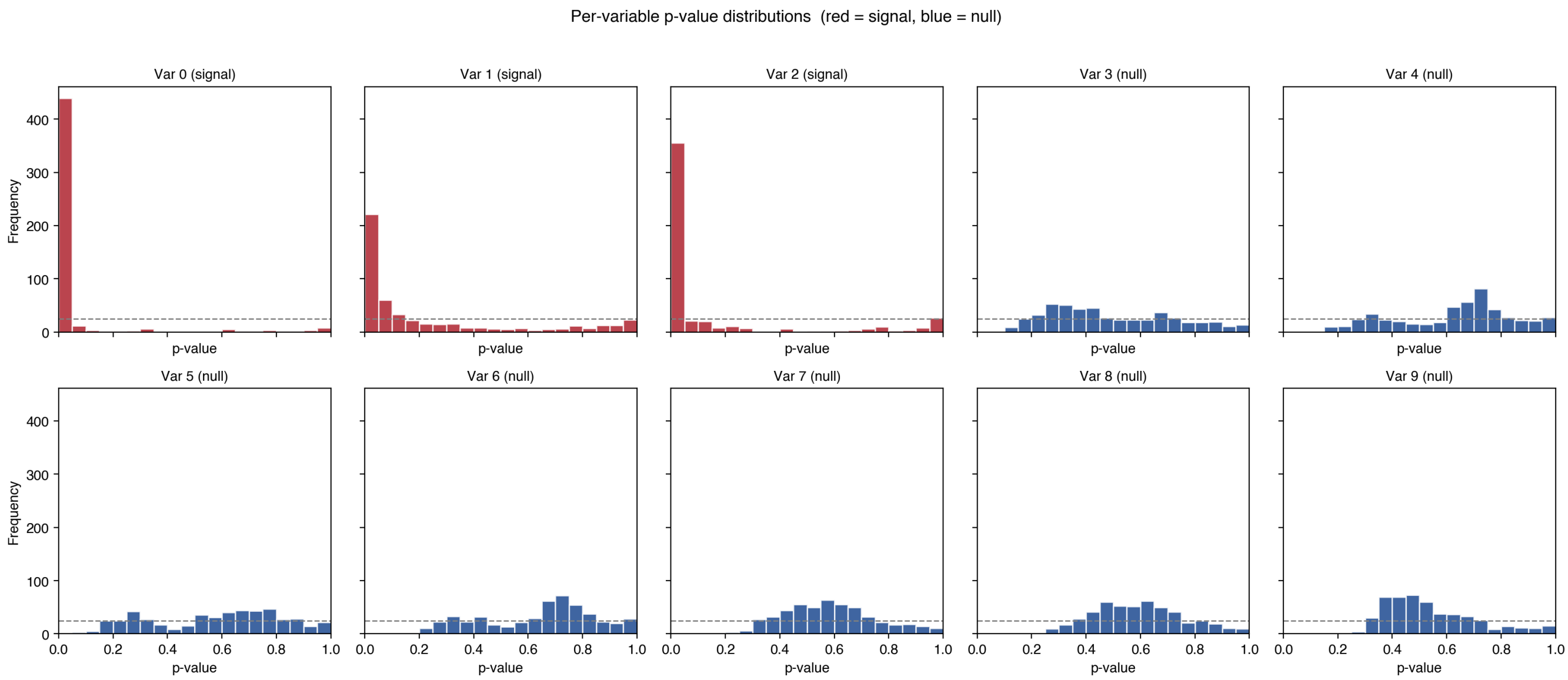

FIGURE S1 Per-variable p-value distributions over 500 repetitions of the permutation test, with signal target j_3 and visit-sample size $V = 5$. Red panels: variables that carry the true context pattern (j_1, j_2, j_3). Blue panels: data-generating-process-null variables (3–9). The gray dashed line marks the expected count per bin under a uniform distribution on $[0, 1]$. Empirical Type-I rate at $\alpha = 0.05$ is 0.000 across all seven null variables.

S2 | SENSITIVITY TO THE VISIT-SAMPLE SIZE V

The permutation test of Section 2.3 samples V visit observations $\mathbf{x}_v^{\text{visit}}$ when the full p^V enumeration is infeasible. The choice of V trades off the resolution of the empirical null against computational cost and could, in principle, bias inference. We therefore swept $V \in \{5, 6, 7\}$ on the same trained model used in Appendix S1, with full enumeration of the p^V row-selection permutations per repetition and 500 repetitions per setting.

Results for both simulations are shown together in Figure S3 (top row: synthetic simulation; bottom row: PBC2-substrate simulation). On the synthetic simulation, signal variables show a monotonically decreasing mean p-value with V (e.g. from 0.057 to 0.027 for j_1, and from 0.162 to 0.122 for j_3), and the rejection rate at $\alpha = 0.05$ correspondingly increases. Larger V therefore confers more power to detect true context effects. Null variables show a rejection rate of exactly 0.000 at every V, across all seven null variables and 500 repetitions. Larger V does not inflate Type-I error in this simulation.

In this simulation, a larger V is therefore strictly beneficial: more power on the real signal, no cost on the null.

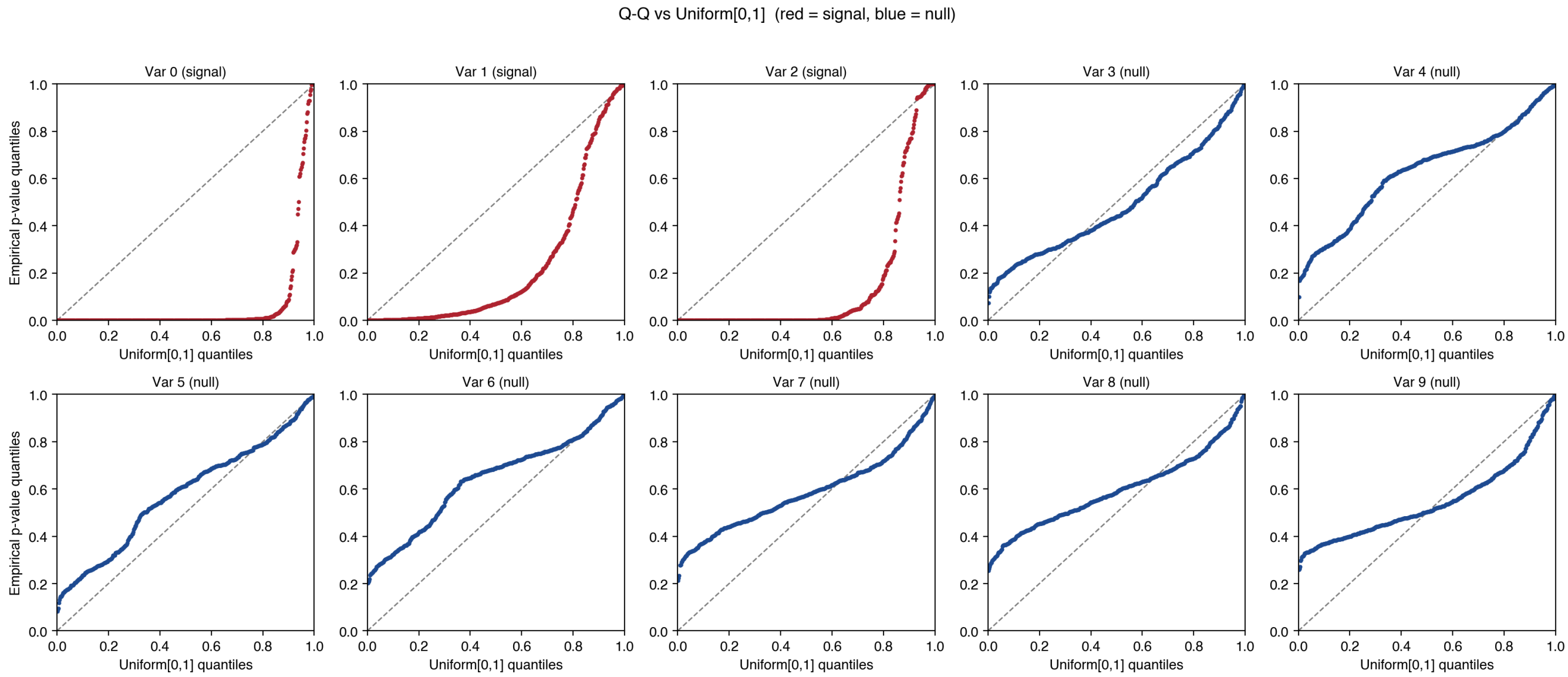

FIGURE S2 Quantile-quantile plots of the empirical p-value distributions in Figure S1 against Uniform[0, 1], for all ten variables. Red panels: data-generating-process signal variables (j_1, j_2, j_3); the empirical quantile curve falls well below the 45° line, indicating an excess of small p-values (i.e. power). Blue panels: data-generating-process null variables (3–9); the empirical quantile curve sits systematically above the 45° line, the conservative shift predicted by the analysis of Section 2.3.

PBC2-substrate simulation.

We repeat the V-sweep on the controlled PBC2-substrate simulation of Section 3.1 (the real binarized PBC2 predictor sequences with the synthetic $j_1 \to j_2 \to j_3$ target overwriting the ascites column; triggers bili_high and albumin_low). A single MiniTransformer is trained on the full substrate ($H = 8$ heads, $C = 8$ cumulants, 150 epochs) and the permutation test is run at the target index for $V \in \{5, 6, 7\}$ with 500 repetitions. As V grows, both signal variables become *more* significant (bili_high: mean p from 0.075 to 0.035; albumin_low: mean p from 0.192 to 0.124), while all seven null-by-construction predictors stay flat and conservative (mean $p \approx 0.53$–0.63, rejection rate ≈ 0) at every V (Figure S3, bottom row). Despite carrying the real correlation structure of the PBC2 variables, the substrate shows no drift of null predictors toward significance: the V-dependent pathology does not materialize, and the significance ranking sharpens rather than collapses as V increases. Whether this monotonicity continues to hold on real cohort data with many small effects (as opposed to the known signal/null structure of these simulations) is examined directly on the LORA cohorts next.

For the LORA cohort, we repeat the same V-sweep on the LORA Dataset 1 and Dataset 2 of Section 3, where ground-truth labels for signal and null variables are not available. The aim is to verify that the qualitative behavior observed on the simulations continues to hold on real cohort data of the size and type targeted by the method, and in particular to ask whether increasing V pushes all candidate context variables toward significance, a concern that motivates examining the test across a range of V. For each of the two LORA datasets, we use the same architecture and training schedule as in Section 3. Using the trained model to predict the GHQ target on each dataset, we ran the permutation test for $V \in \{5, 6, 7\}$ with 500 repetitions. Results for both datasets are shown in Figure S4 (top row: Dataset 1; bottom row: Dataset 2).

The qualitative pattern matches the simulations above: variables that the model has learned to use as informative contexts for the GHQ target, on Dataset 1, these are dh_38 (Housekeeping) and dh_45 (Noise), in agreement with the ranking in Table 5, show monotonically decreasing mean p-values and increasing rejection rates at $\alpha = 0.05$ as V grows. The top-ranked context on Dataset 1, Housekeeping, has an empirical rejection rate at $\alpha = 0.05$ rising from 0.914 at $V = 5$ to 0.982 at $V = 7$. Variables that were not flagged as significant at the smallest V stay flat or, for several variables, become *more* conservative as V grows (e.g. le_8 on Dataset 1: mean p $0.780 \to 0.818$ as V goes from 5 to 7). On Dataset 2, the top-ranked contexts identified at $V = 7$, dh_38, dh_46, le_17, dh_37, overlap closely with the paper's Table 5 ranking, and the same monotonic separation between flagged and unflagged variables is observed.

In neither dataset do we see all candidate contexts being driven toward significance as V grows. Instead, larger V amplifies the separation between contexts the model has actually learned to rely on and contexts it has not. This is consistent with the

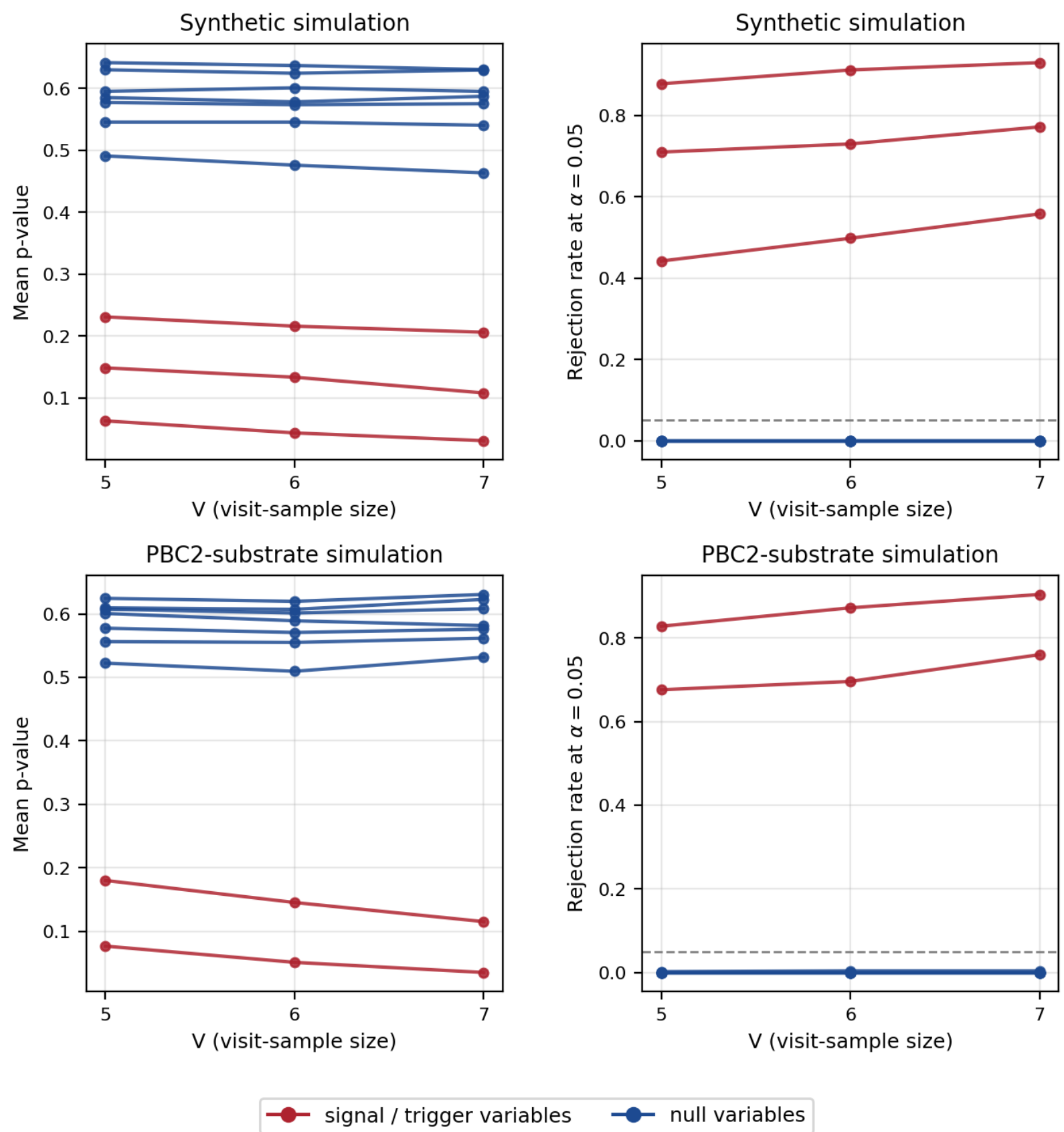

FIGURE S3 Sensitivity of the permutation test to the visit-sample size V and 500 repetitions. Top row: synthetic simulation; signal variables are j_1, j_2, j_3. Bottom row: PBC2-substrate simulation; the two generative triggers are bili_high and albumin_low. Left column: mean p-value per variable. Right column: empirical rejection rate at $\alpha = 0.05$ (gray dashed line). Red lines: signal variables. Blue lines: null variables; their rejection rate sits on the $y = 0$ axis at every V in both simulations.

simulation finding that the test's null behavior is conservative even when the empirical null is contaminated, and provides empirical evidence on real cohort data that the choice of V does not by itself drive the significance ranking.

S3 | BASELINE COMPARISON AND PARAMETER MATCHING

This appendix gives the details of the prediction comparison summarized in Section 3. We compare MiniTransformer against four compact-transformer baselines, in addition to the existing average/regression/carry-forward baselines using the same training setting.

Architectures.

- **iTransformer**[35] inverts the attention axis: each variable's full history is projected to a d_{model}-dimensional token, and attention operates across the p variable-tokens rather than across time steps. Applied at each prediction position using a causal history window of length $L = 10$; feed-forward width $2d_{\text{model}}$, 1 head; d_{model} chosen to match MiniTransformer's parameter count.
- **Scaled-down vanilla transformer**[1] 1 encoder layer, 1 attention head, causal self-attention mask, learned positional embeddings, feed-forward width $4d_{\text{model}}$; d_{model} chosen to match MiniTransformer's parameter count.
- **DLinear**[8] decomposes each channel's history (window $L = 10$) into trend (moving average, kernel size 5) and remainder; applies a separate per-channel linear map to each component and sums the predictions. No cross-channel mixing or attention.
- **RoPEAttention**[36] MiniTransformer with the temporal-decay positional scheme replaced by rotary position embeddings on the per-head Q/K projections; the prediction-horizon decay is also removed, giving uniform cumulant pooling. RoPE

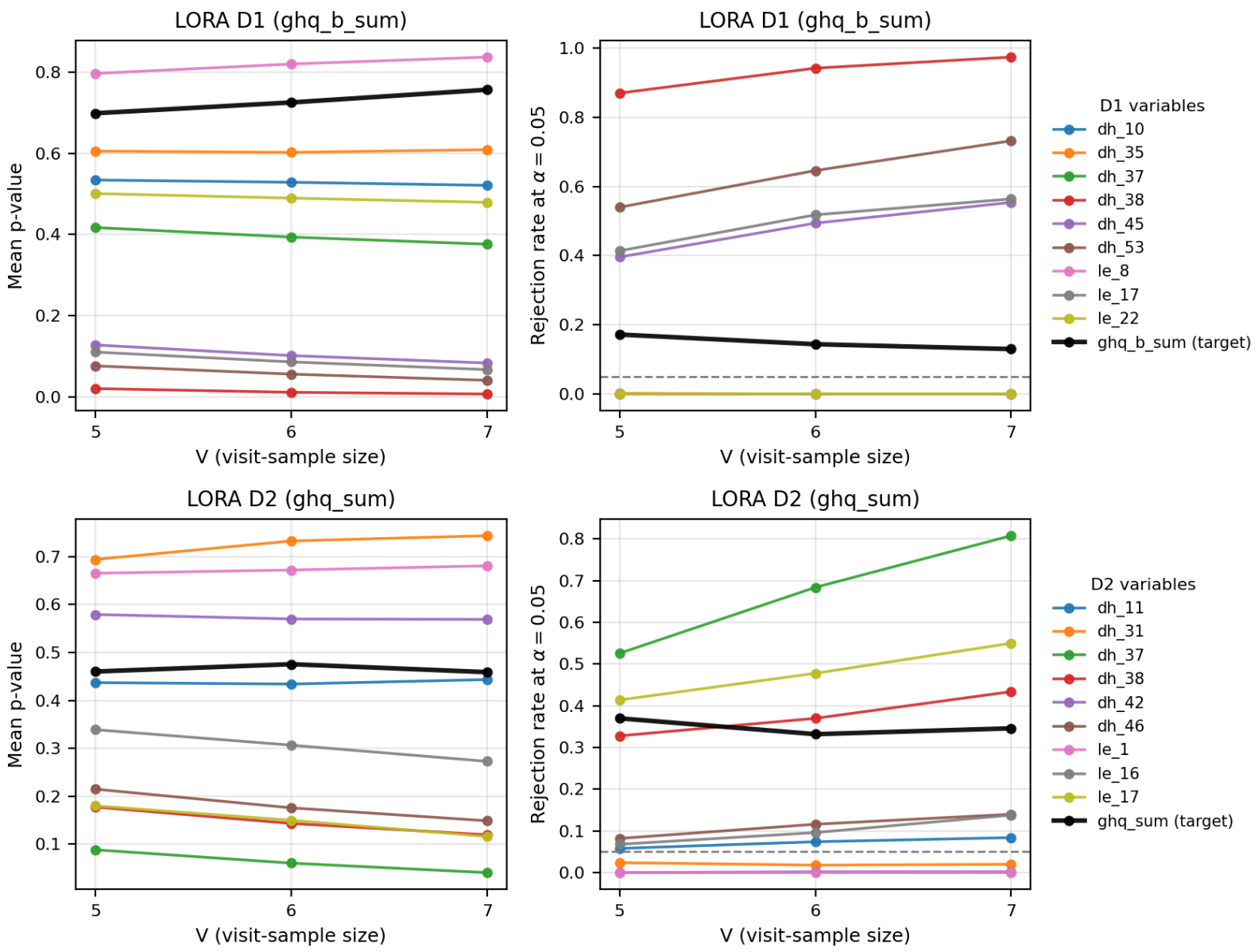

FIGURE S4 Sensitivity of the permutation test to the visit-sample size V on the LORA cohorts, fold 0, and 500 repetitions. Top row: Dataset 1 (ghq_b_sum); bottom row: Dataset 2 (ghq_sum). Left column: mean p-value per candidate context. Right column: empirical rejection rate at $\alpha = 0.05$ (gray dashed line). Each colored line is one candidate context variable, labeled by its code (matching Table 5); the bold black line is the GHQ target variable shown as a reference. On both datasets, the contexts the model has learned to rely on become more significant with V, while the remaining contexts stay flat or become more conservative.

requires $d_k \geq 2$ (rotation acts in 2D subspaces), which breaks the scalar-attention structure that makes Eq. 5 a closed-form expression; this variant is therefore included as a baseline rather than as a positional-scheme ablation of MiniTransformer.

Parameter matching.

The trainable parameter counts are 420–452 for the MiniTransformer, 378 for the scaled-down vanilla transformer, 427 for the iTransformer, 594–714 for RoPEAttention, and 220 for DLinear (the ranges span the simulation configuration with 12 heads and 2 cumulants and the real data configuration with 8 heads and 8 cumulants; the other baselines are sized by d_{model} and are identical in both settings). We tune the width of the scaled-down vanilla transformer and the iTransformer to match the MiniTransformer's count as closely as the architecture allows. Exact matching is not possible for the remaining two: DLinear consists only of per-channel linear maps and is therefore intrinsically smaller, while RoPEAttention requires $d_k \geq 2$ (rotation acts in 2D subspaces) and is therefore intrinsically larger than MiniTransformer's scalar attention. These two consequently bracket the MiniTransformer in size rather than matching it exactly, which we note so that the comparison is read with the parameter budget in mind.

All models are trained and evaluated under identical conditions. On the simulation, each model is run over 10 random seeds ($\{0, 1, 11, 42, 123, 999, 1337, 2025, 9999, 12345\}$) with $n_{\text{test}} = 1000$ held-out sequences per seed, for each training size $n_{\text{train}} \in \{100, 200, 500, 1000\}$. On LORA Dataset 1, LORA Dataset 2, and the PBC2-substrate simulation, we use 10-fold cross-validation with one seed per fold from the same list. The same seeds and folds are used for every model, so the comparison is paired. Full per-seed and per-fold numbers, together with the code to regenerate them, are available in the repository linked in Section 3.

Table 1 of the main text reports the mean ± standard deviation across seeds (simulation) or folds (cohorts). Two patterns are worth noting. First, on the LORA cohorts the four compact-transformer baselines, the MiniTransformer, and the regression baseline are tightly clustered on MSE_{tar}, differing by at most about 0.02 across the 10-fold mean, with the gaps lying within the fold-to-fold standard deviation. In this regime, the architectural simplifications of the MiniTransformer neither penalize nor advantage prediction relative to comparably sized models, and its distinctive contribution is instead the closed-form context-effect statistic of Section 2.3 together with the interpretable temporal-decay parameters. Second, on the controlled simulation the parameter-matched scaled-down vanilla transformer attains the lowest MSE_{j_3} across all training sizes, since the sharp $j_1 \to j_2 \to j_3$ chain is precisely the regime in which additional capacity can reach the optimum; the MiniTransformer is

consistently the second-best model there and the best on the PBC2-substrate simulation, where the predictors carry realistic correlation structure.

S4 | DECAY AND CUMULANT-HEAD ABLATIONS

This appendix reports three ablations that isolate the contributions of the two temporal-decay terms and of the cumulant pooling itself, all trained in a single common protocol so their magnitudes are directly comparable. The *PairwiseDecayOff* variant sets the pairwise temporal-decay multiplier in the attention kernel (1) to 1, leaving standard softmax attention while retaining the full cumulant pooling; this isolates the contribution of the pairwise temporal-decay kernel. The *HorizonDecayOff* variant retains the cumulant pooling but sets the prediction-horizon decay factor of (3) to 1, so that history is pooled uniformly toward the prediction step. The *CumulantOff* variant removes the cumulant head entirely, using the per-timestep output of (2) at $t = T$ as the readout. Each variant differs from the MiniTransformer by exactly one frozen scalar (w_{dist} for PairwiseDecayOff, $w_{horizon}$ for HorizonDecayOff and CumulantOff), Thus, any observed differences can be attributed to the ablated term rather than to a wholesale architectural change. All variants are trained with the same protocols as the main text (10 seeds on the simulation, 10-fold cross-validation on the cohort settings; the PBC2 column uses the synthetic-target setup of Section 3.1.2).

Table S1 reports $\text{MSE}_{\text{target}}$ for all three ablations alongside the full MiniTransformer. On the two settings with predictive headroom, the synthetic simulation and the PBC2-substrate simulation, a consistent ordering emerges. Removing either decay term costs a moderate and comparable amount, with the horizon decay slightly more impactful than the pairwise decay: on the simulation, MSE_{j_3} rises from 0.106 to 0.141 when the pairwise decay is removed and to 0.156 when the horizon decay is removed; on the PBC2-substrate simulation it rises from 0.097 to 0.107 and 0.111 respectively. Removing the cumulant head entirely is in a different class: MSE_{j_3} jumps to 0.372 on the simulation, of the same order as the marginal-mean baseline, and to 0.131 on the PBC2 substrate. On the two LORA cohorts, where there is little predictive headroom over the marginal-mean baseline, all three ablations are within seed-level noise of the full model.

TABLE S1 Decay and cumulant-head ablations on $\text{MSE}_{\text{target}}$, all trained in the same protocol on a common baseline. PairwiseDecayOff sets the pairwise temporal-decay multiplier of (1) to 1 (standard softmax attention), retaining the cumulant pooling. HorizonDecayOff sets the prediction-horizon decay of (3) to 1 (uniform causal pooling). CumulantOff removes the cumulant head entirely, using the per-timestep output of (2) at $t = T$. Mean ± standard deviation across 10 seeds (simulation) or 10 folds (cohorts). The PBC2 column uses the synthetic-target setup of Section 3.1.2 (real binarized predictors, the ascites column overwritten by the $j_1 \to j_2 \to j_3$ rule).

Model	Sim. (j_3, $n_{\text{train}} = 200$)	LORA Dataset 1	LORA Dataset 2	PBC2 sim. (synthetic y)
MiniTransformer	0.106 ± 0.017	0.186 ± 0.020	0.183 ± 0.024	0.097 ± 0.018
PairwiseDecayOff	0.141 ± 0.006	0.190 ± 0.018	0.182 ± 0.024	0.107 ± 0.027
HorizonDecayOff	0.156 ± 0.009	0.193 ± 0.020	0.182 ± 0.023	0.111 ± 0.034
CumulantOff	0.372 ± 0.024	0.192 ± 0.028	0.190 ± 0.025	0.131 ± 0.042

As discussed in Section 2.2, the pairwise decay (Eq. 1) shapes attention within the history while the horizon decay (Eq. 3) controls each visit's contribution to the cumulative sum; the ablations in Table S1 quantify how much each term matters. Removing the horizon decay (HorizonDecayOff) collapses near and distant history to equal weight in the cumulative sum, losing the temporal proximity prior for prediction. Neither term dominates, and removing either degrades the model where there is sharp temporal structure to exploit. The cumulative pooling itself carries most of the predictive value: the per-timestep output of (2) at $t = T$ alone (CumulantOff) does not retain the information aggregated by the pooled cumulant, even though it is built from the same per-timestep representations. The cumulant head is therefore the stage at which the horizon-axis prior enters and at which evidence is aggregated across the history. The neutrality of the ablations on the LORA cohorts does not diminish this role: a neutral ablation result means the ablated component was not predictively necessary on that cohort but this is still an interpretable parameter showing the effect of history.

S5 | FULL-SIZED TRANSFORMER VS MINITRANSFORMER

We trained two models on the two LORA datasets (Dataset 1: ghq_b_sum, n = 882 individuals, p = 10 binarized stressors, GHQ-b target; Dataset 2: ghq_sum, n = 878 individuals, GHQ target):

- a deliberately over-parameterized vanilla transformer with four encoder layers, $H = 4$ attention heads, $d_{\text{model}} = 128$, $d_{\text{ff}} = 512$, dropout 0.1; this gives 797 066 trainable parameters, roughly 900 parameters per training individual on each cohort;
- the paper's MiniTransformer, with $H = 8$ scalar attention heads, $C = 8$ cumulants, $d_k = d_v = 1$, totalling 420 parameters.

Both models share the rest of the training protocol: 150 epochs, Adam optimizer at learning rate 10^{-3}, mean-squared-error loss on the next observed time step. We use the same 10-fold cross-validation split as Section 3 and for each fold we record the per-epoch training MSE and validation MSE on the chosen GHQ target. Trajectories are then aggregated across folds and reported as mean ± 1 standard deviation.

The full-sized transformer overfits in the textbook way on both datasets (Figure S5, red curves). Training MSE decreases monotonically toward zero; validation MSE on the target bottoms out very early and then climbs steadily. On Dataset 1 the fold-averaged minimum is 0.192 ± 0.022 at epoch 8, climbing to 0.285 ± 0.034 at epoch 150 (a rebound of +0.093). On Dataset 2 the same shape repeats: minimum 0.185 ± 0.018 at epoch 7, climbing to 0.257 ± 0.038 at epoch 150 (a rebound of +0.072). No double-descent recovery occurs within the training window on either dataset. The same protocol with MiniTransformer behaves very differently (Figure S5, blue curves). Validation MSE on the target descends gradually and flattens. On Dataset 1, the fold-averaged minimum is 0.183 ± 0.020 at epoch 137, and the value at epoch 150 is 0.186 ± 0.020. On Dataset 2, the fold-averaged minimum is 0.184 ± 0.021 at epoch 124, and the value at epoch 150 is 0.186 ± 0.023. Effectively, no overfit occurs in the training window on either dataset; the training and validation curves remain close throughout.

Even at the over-parameterized transformer's *oracle-best* early-stopping epoch, its fold-averaged validation MSE is essentially no better than MiniTransformer's fold-averaged minimum (Dataset 1: 0.192 vs 0.183; Dataset 2: 0.185 vs 0.184), despite using roughly 1 900 times more parameters. At any natural training horizon (i.e. when training is not stopped at the validation-set minimum, which is not in general a valid model-selection protocol) the over-parameterized transformer substantially underperforms MiniTransformer (Dataset 1: 0.285 vs 0.186; Dataset 2: 0.257 vs 0.186). This is consistent with the position that benign overfitting and double-descent phenomena do not transfer to the $N \sim 10^2$, $T \sim 10$ longitudinal cohort regime that motivates the MiniTransformer architecture.

S6 | SENSITIVITY TO THE KERNEL-SHAPE PARAMETER γ

The temporal-decay kernel of Eq. (1) and the prediction-horizon decay of Eq. (3) both use a shape parameter γ that the main text fixes to $\gamma = 5$. To assess sensitivity to this choice, we re-trained the MiniTransformer with $\gamma \in \{1, 2, 5, 10\}$, and additionally with γ treated as a free scalar parameter learned from the data (initialized at $\gamma = 5$). Training protocols match the corresponding main-text analyses: ten random seeds on the simulation (matching Section 3.1), and ten-fold cross-validation on LORA Dataset 1 and LORA Dataset 2 (matching Section 3). Table S2 reports the resulting $\text{MSE}_{\text{target}}$ across the three datasets.

On the simulation, performance is stable for $\gamma \in \{1, 2, 5\}$, with $\gamma = 5$ best on the mean and $\gamma = 10$ over-sharpening the kernel and degrading MSE by roughly 1σ. On the LORA cohorts, the picture is flatter still: MSE varies by less than 0.001 across the four fixed-γ choices on both Dataset 1 and Dataset 2. The learned-γ variant attains essentially the same MSE as $\gamma = 5$ on every dataset, with the learned value averaging 4.35 across simulation seeds, 1.34 across Dataset 1 folds and 1.83 across Dataset 2 folds. The simulation's much higher learned γ reflects that the sharp $j_1 \to j_2 \to j_3$ triggers built into the synthetic data favor a more peaked decay kernel than the smoother temporal dynamics of the real cohorts. Overall, MiniTransformer's predictive behavior is essentially invariant to the choice of γ in our regime: the paper's fixed $\gamma = 5$ is reasonable for the controlled-simulation regime but is not pivotal, and the learned-γ variant offers a parameter-free alternative when the analyst does not wish to fix the kernel shape *a priori*.

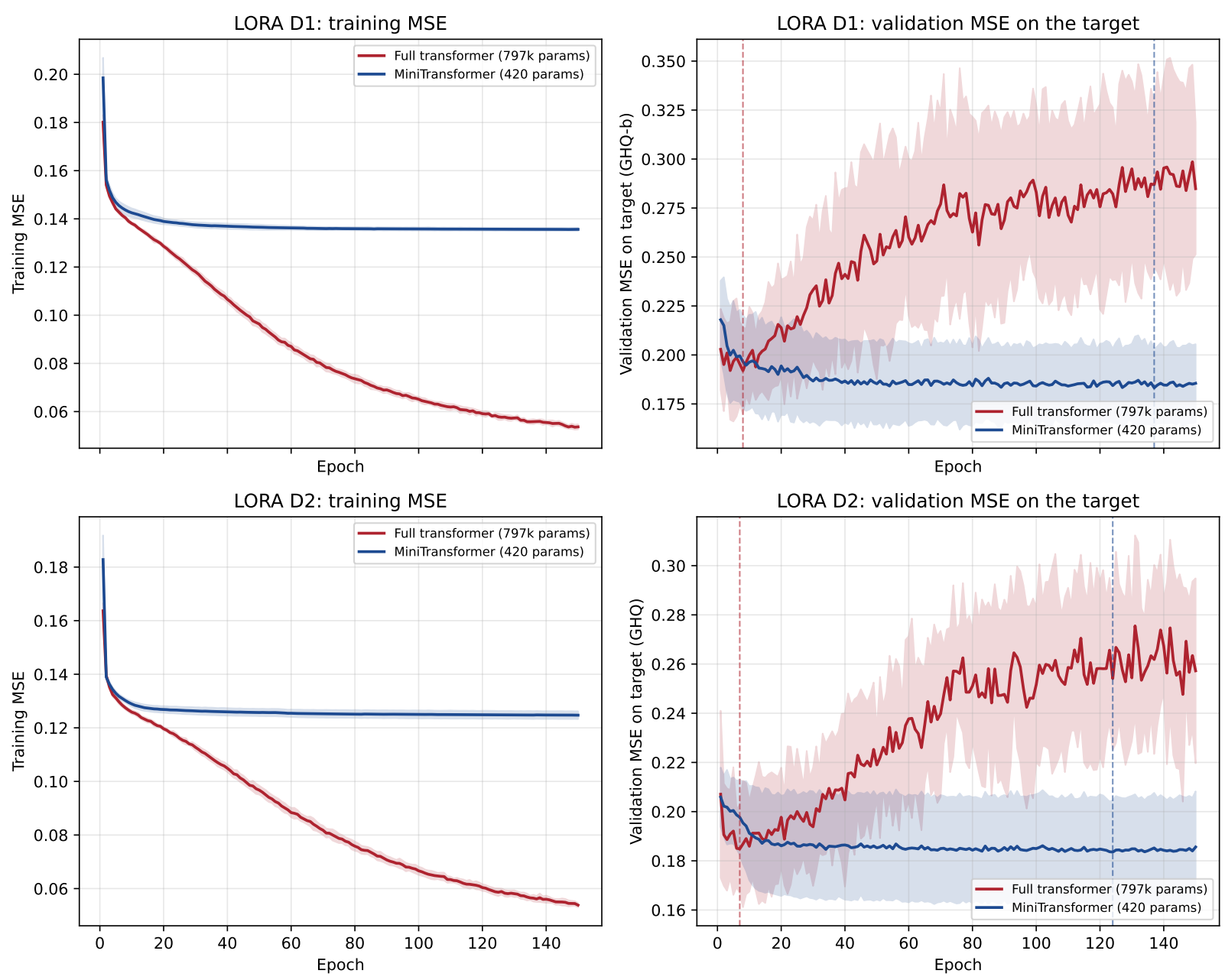

FIGURE S5 Training (left column) and validation MSE on the target (right column) on LORA Dataset 1 (top row) and LORA Dataset 2 (bottom row), 10-fold cross-validated, 150 epochs per fold, all models trained with the canonical per-fold seeds. The over-parameterized vanilla transformer ($\sim$ 797 000 parameters; red) overfits sharply on both datasets: validation MSE on the target reaches its minimum at epoch 8 on Dataset 1 (0.192 ± 0.022) and epoch 7 on Dataset 2 (0.185 ± 0.018), then climbs steadily to 0.285 ± 0.034 and 0.257 ± 0.038 respectively by epoch 150, with no double-descent recovery in the window. The paper's MiniTransformer (420 parameters; blue) does not overfit: its validation MSE descends gradually to 0.183 ± 0.020 at epoch 137 on Dataset 1 and 0.184 ± 0.021 at epoch 124 on Dataset 2, and stays effectively flat thereafter. Solid lines: 10-fold mean. Shaded bands: ± 1 standard deviation across folds. Dashed vertical lines mark the fold-averaged validation-minimum epoch for each model.

TABLE S2 γ-sensitivity on the simulation (10 seeds, $n_{\text{train}} = 200$) and on LORA Dataset 1 and Dataset 2 (10-fold CV). Mean $\pm$ std of $\text{MSE}_{\text{target}}$. The "learned" row treats γ as a free scalar parameter, initialized at $\gamma = 5$; its mean learned value across seeds or folds is shown in the last row.

γ	Synthetic simulation (j_3)	LORA Dataset 1	LORA Dataset 2
1	0.123 ± 0.024	0.181 ± 0.035	0.179 ± 0.016
2	0.117 ± 0.041	0.181 ± 0.034	0.179 ± 0.016
5	$\mathbf{0.109 \pm 0.038}$	0.182 ± 0.036	0.181 ± 0.015
10	0.137 ± 0.008	0.182 ± 0.034	0.182 ± 0.019
learned	0.109 ± 0.038	0.181 ± 0.034	0.179 ± 0.016
mean learned γ	4.35	1.34	1.83
